# Danshen-Honghua Ameliorates Stress-Induced Menopausal Depression in Rats

**DOI:** 10.1155/2018/6589608

**Published:** 2018-05-03

**Authors:** Simeng Gu, Yao Ma, Kemin Ge, Ruifang Nie, Erxi Wu, Yang Li

**Affiliations:** ^1^Department of Psychology, Jiangsu University Medical School, Zhenjiang 212013, China; ^2^School of Life Science, Nanjing University of Chinese Medicine, Nanjing 210023, China; ^3^Department of Neurosurgery, Baylor Scott & White Health, Temple, TX 76358, USA; ^4^Department of Surgery, College of Medicine, Texas A&M University, Temple, TX 76354, USA; ^5^Department of Pharmaceutical Sciences, College of Pharmacy, Texas A&M University, College Station, TX 77843, USA; ^6^School of Psychology, Nanjing Forest Police College, Nanjing 210023, China

## Abstract

**Objective:**

Previously, we have shown that Danshen-Honghua (DSHH) for cognitive deficits after ischemia induced impairments of the hippocampus. Here, we investigate the effects of DSHH on stress-induced depression in menopausal rats.

**Methods:**

A rat model with menopausal depression was established with bilateral ovariectomies in female SD rats followed by chronic mild stress treatment for 21 days. 40 rats were randomly divided into the sham surgery group (sham surgery and no stress treatment), surgery group (surgery with no stress treatment), surgery/stress group (surgery and stress treatment), fluoxetine group (2.4 mg·kg^−1^, with surgery and stress treatment), and DSHH group (35 g·kg^−1^, with surgery and stress treatment). The rats in the last two groups were treated with stresses together with intragastric drug administration for three weeks after the surgery. Then open-field locomotor scores and sucrose intake were tested for behavior changes. Also, the levels of norepinephrine (NE), dopamine (DA), serotonin (5-HT), and cortisone were determined by high-performance liquid chromatography (HPLC). Serum estradiol (E_2_), follicle-stimulating hormone (FSH), and luteinizing hormone (LH) were determined by radioimmunoassay.

**Results:**

The results of open-field locomotor scores, sucrose intake in both the fluoxetine group and DSHH group, were significantly higher than those of the surgery/stress group (*P* < 0.01). Serum LH, FSH, and cortisone levels in both the DSHH group and fluoxetine group were significantly lower than those in the surgery/stress group (*P* < 0.01). Serum E_2_ levels in these groups were slightly increased in these medicine groups (*P* < 0.01). The monoamine levels in the DSHH group were much higher than those in the surgery/stress group (*P* < 0.01).

**Conclusion:**

DSHH can ameliorate stress-induced depressed syndromes in the surgery/stressed rats via regulating LH and FSH levels as well as monoamine levels.

## 1. Introduction

Major depressive disorder (MDD) is a leading health-related cause of human suffering [1, 2]. The pathological mechanisms of MDD are far from clear [2, 3] and are a hot topic for neuroscience research [4, 5]. Previous studies have pointed out that the mechanism of depression is majorly due to the monoamine neurotransmitters, including norepinephrine (NE), serotonin (5-HT), and dopamine (DA) [6, 7], because most of the first choice antidepressants affect 5-HT and NE or 5-HT reuptakes. However, even though many studies support the role of the central monoamine network, other hormones or neuromodulators are involved too, such as hormones released from the hypothalamus-pituitary axis (HPA) [8] and sex hormones [9]. Depression occurrence in female is twice as many as male patients [10] and is a well-known symptom for the menopausal period [11, 12]. In fact, one of the major symptoms of menopause is depression [12, 13]. However, the treatment methods for menopausal depression are different. A published study from our colleagues showed that a Chinese medicine, Danshen-Honghua (DSHH), is effective for cognitive deficits after ischemia-induced impairments of the hippocampus [14]. A wealth of clinical experience has demonstrated that DSHH is very effective in the treatment of menopausal depression. Here, we tested its mechanisms in the treatment of depression by screening many hormone and neurotransmitter changes after administration with these drugs. This study will not only shed light on the mechanisms of depression but also help us find alternative ways for depression treatment.

## 2. Materials and Methods

### 2.1. Animals

40 female Sprague-Dawley (SD) rats with body weight averaging 500 ± 20 g, at the age of 12 months, were randomly divided into 5 groups: the sham surgery group (sham surgery and no stress treatment), surgery group (surgery with no stress treatment), surgery/stress group (surgery and stress treatment), fluoxetine group, and DSHH group, with 8 rats in each group. The animals were treated as shown before: the sham surgery group rats were treated with sham surgery (only the skin was opened), the surgery group rats were treated with surgery to remove the ovaries but no stress treatment was introduced, the surgery/stress group rats were treated with surgery and stress, the fluoxetine group rats were treated with fluoxetine (2.4 mg·kg^−1^) in addition to surgery and stress, and the DSHH group rats were treated with DSHH (35 g·kg^−1^) in addition to surgery and stress. The rats in the last two groups were treated with stresses together with intragastric drug administration for three weeks continuously, three times a day, after the surgery recovery. DSHH included *Carthamus tinctorius* 15 g and *Salvia miltiorrhiza* Bge 20 g. These drugs were grounded into powder and mixed with 200 mL deionized water and intragastrically administrated according the 35 g·kg^−1^ amounts. All the procedures were approved by the Institution of Animal Care and Use Committee.

## 3. Instrument

US Thermo Microplate Reader and KH30R desktop high-speed refrigerated centrifuge were used in the experiments. Analytical balance, low-temperature ultracentrifuge, paraffin embedding machine, slicer, and so on are provided by the central laboratory. 3200 ATRAP high-performance liquid chromatography (HPLC) tandem mass spectrometer (ABI, USA), equipped with atmospheric pressure chemical ionization sources (LC-APCI-MS/MS).

## 4. Animal Surgery/Control

### 4.1. Ovary Removal [2]

In addition to the sham surgery group, the other four groups were given “ovariectomy.” During the surgical procedure, the rats were given 100 mg·kg^−1^ ketamine to induce anesthesia and fixed on a hard plate in supine position. A marker was marked in the rat 2 cm outside the spine and 1 cm below the ribs; the soft tissue was sectioned separately. The peripheral blood vessels were ligated, and both sides of the ovaries were removed, and finally, the uterus was removed. The method of determining successful ovariectomy is the vaginal epithelium keratosis test: 5 days of continuous monitoring of the rat vagina did not find the estrous cycle. The animals were screened with the estrous cycle one week before the surgery, to make sure they have normal cycles.

### 4.2. Chronic Mild Unpredictable Stress

In addition to the sham surgery group and the surgery group, the other three groups of rats were housed in a single cage after the surgery and after 5 days of surgery, they were treated with chronic unavoidable stresses for 21 days. Stress treatment lasted for 2 h, including 36 V AC electric foot shock (stimulated 1 time every l min; each time lasted 10 s, for a total of 30 times), 4°C ice-cold water swimming (5 min), 45°C heat stress (5 min), 15 min shaking (1 times/s), 45-degree tilting of the rat cage (24 h), tail clipping (1 min), bed wetting (10 h), bottle emptying (1 h), and application of each stimulus 2 times.

## 5. Administration

Rats in each group, except the sham surgery group, were treated consecutively with stress for 21 days, after the surgery recovery. Rats in the fluoxetine group were given Prozac 2.4 mg·kg^−1^, 1 time per day. Rats in the DSHH group were given DSHH 35 g·kg^−1^, which was divided 2 times a day through gavage (the dosage was referred to a previous publication [14]). In the course of study, two rats died—one in the surgery/stress group and one in the fluoxetine group died during intragastric administration. Because these two rats were dead by accident (not related to the treatments from this study), they were excluded from the number of studies. On the last treatment day, all 38 rats were sacrificed (cervical dislocation). Each rat was rapidly decapitated and 5–10 mL of blood was obtained. The serum was separated by centrifuge.

## 6. Observation Indicators

### 6.1. Open-Field Test

Open-field test was done in an open box with both the length and width to be 80 cm and the height to be 40 cm, the same as those that we have reported before [15]. The bottom surface was divided into 25 large areas with white lines. The rats were placed at the center of the bottom of the box, and the vertical activity points were measured as the number of uprights, from the time the rat's feet left the bottom to the time their feet back to the bottom. The level of activity of rats was measured through the number of bottom blocks, through 1 grid for 1 point, such as rats walking along the line, every 10 cm considered as 1 time. Each rat walks one time for 3 min.

### 6.2. Sucrose Intake Test

Sucrose intake test was carried out on the 21st day after surgery recovery. Each rat was given 135 mL of 1% sucrose solution after 24 h fasting, and the amount of sucrose solution consumed by rats was calculated.

## 7. Sex Hormone Measurement

Estradiol (E_2_), luteinizing hormone (LH), and follicle-stimulating hormone (FSH) were measured. On the last treatment day, the abdominal aorta was taken and the serum was separated as soon as possible (within 8 h). The contents of E_2_, LH, and FSH were determined by radioimmunoassay. The RIA kit used was bought from the United States Depp Company. Specific operation was carried out according to the instructions.

## 8. Measurement of Monoamine Neurotransmitters

After the chronic stress test and the behavior test, the animals were decapitated under anesthesia with isoflurane (2%). The brain was quickly removed and the cortex and hippocampus were collected for HPLC and electron microscopy experiment. The cerebral spinal fluid was saved in a glass syringe and injected into a HPLC to measure NE, DA, and serotonin. The monoamine levels were assessed by comparing the reference standard and respective peak area and elution time of the samples using a calibration curve for each monoamine neurotransmitter.

## 9. Statistical Processing Method

SPSS20.0 software was used for statistical analysis, and the data were used in the form of $\bar{x}\pm s$. The data were compared with *t*-test; analysis of variance was used to compare the difference between groups.

## 10. Results

### 10.1. Body Weight

Table 1 shows that there was no significant difference in body weight among the five groups before ovariectomy (one-way ANOVA, *P* > 0.05). After surgery and/or stress, the body weights of the animals in each group decreased than those in the sham surgery group and the body weights of the rats in the surgery/stress group were significantly lower than those in the surgery group (one-way ANOVA, ^∗^*P* < 0.01, *N* = 38; Table 1), but there was no significant difference among the surgery/stress group, fluoxetine group, and DSHH group (*P* > 0.05).

### 10.2. Behavioral Assessment

The results showed that there was no significant difference in the behavior tests among the surgery groups (one-way ANOVA, *P* > 0.05, Table 2). The scores of vertical movement and horizontal movement in the surgery groups were significantly decreased than those in the sham surgery group, (one-way ANOVA, *P* < 0.01). The scores of vertical movement and horizontal movement in the fluoxetine group were significantly lower than those in the sham surgery group (one-way ANOVA, *P* < 0.01), and the scores of horizontal movement and vertical movement in the DSHH group were also significantly higher than those in the surgery group (one-way ANOVA, *P* < 0.01), but there was no significant difference between the two groups (one-way ANOVA, *P* > 0.05).

### 10.3. Sucrose Intake

The rats in the castration group consumed less sucrose than those in the sham surgery group (one-way ANOVA, ^∗^*P* < 0.01; Table 3). But the sucrose consumption in the DSHH group was significantly higher than that in the surgery/stress group (one-way ANOVA, ^∗^*P* < 0.01), and the sucrose consumption in the fluoxetine group was significantly higher than that in the surgery/stress group (one-way ANOVA, ^∗^*P* < 0.01), but there was no significant difference between the two groups (*P* > 0.05).

### 10.4. Monoamine Neurotransmitters and Hormones

The levels of neurotransmitters norepinephrine (NE), dopamine (DA), and serotonin (5-HT) in the CSF and cortisone in the serum were tested with high-performance liquid chromatography (HPLC). The levels of NE, DA, and 5-HT in the CSF were significantly lower in the surgery groups compared with the sham surgery group, but cortisone levels in the serum in the surgery/stress group were much higher than those in the sham surgery group. The levels of these monoamines in the two drug-treated groups were significantly higher than those in the surgery/stress group (one-way ANOVA, *P* < 0.05; Table 4). Compared with that of the surgery/stress group, the 5-HT level of the DSHH group was statistically significantly higher (one-way ANOVA, *P* < 0.01).

### 10.5. Sex Hormones

The levels of E_2_ in the serum of the two drug-treated groups were not significantly different in the surgery group and surgery/stress group. Compared with that of the surgery/stress group, the E_2_ level of the DSHH group was not statistically significant (one-way ANOVA, *P* > 0.05; Table 5). The levels of FSH in the two groups were lower than those in the surgery group and surgery/stress group. Compared with those in the surgery/stress group, the levels of FSH in the DSHH group were significantly higher (*t* = 5.740, *P* < 0.05). The level of LH in the treatment group was not significantly different from that in the sham surgery group (one-way ANOVA, *P* > 0.05), suggesting that DSHH can restore ovarian function by adjusting the E_2_ and FSH.

## 11. Discussion

There are tons of studies suggesting that depressions are due to monoamine neurotransmitter changes [16–19]; in addition, many other hormones are also involved, such as sex hormones [20]. In this study, we probed into the effects of sex hormone changes in the menopausal depression after treatment with medicines. We used ovariectomized female SD rats to stop the E_2_ release and found that these rats were much easier to get depressed through chronic mild stress. On the contrary to decreases in E_2_ release, LH and FSH levels are greatly increased, possibly due the removal of feedback inhibition of E_2_. In addition, the levels of cortisone are also increased, which suggested that LH and FSH in the pituitary might affect cortisone directly. Consistent with the increase of LH and FSH after ovariectomization in the rats, this phenomenon also exists in the menopause patients. LH, FSH, and ACTH are all released from the pituitary gland, which are possibly interacted with each other. LH and FSH leading to depression might also be the reasons for women being prone to depression, especially LH, whose release surges up during ovulation in the menstrual cycle [12], and LH detection is used to detect ovulation, which occurs about 24–48 hours after the LH surge. In all, LH release increase might be the reason for depression at both the ovulaton in the menstrual cycle and menopause.

Contrary to enhancing E_2_, which might have a negative feedback on LH release after menopause and thus depression, DSHH can reduce the LH release after ovary removal, increase CSF monoamine concentrations, and improve the score of horizontal movement and vertical movement effectively in the surgery rats. In addition, DSHH increased the consumption of sugar water, suggesting that DSHH can alleviate depressed syndromes in menopausal depressive rats via regulating hormone levels, especially LH. In this experiment, the activity of the rats in each group was reflected by the horizontal activity score [21]. The sensitivity of rats to the reward substance was reflected by the consumption of sugar [2]. After a period of stress, the score of exploration behavior, activity level, and sugar consumption in the surgery/stress groups significantly decreased. These data suggested that the DSHH can successfully reverse LH surge after ovary removal and also increase the monoamine neurotransmitters in the menopausal rats with the lack of interest, loss of will behavior, loss of pleasure, and so on. Ingredients of DSHH such as Danshen and Honghua support the synergistic effects on promoting blood circulation and removing blood stasis [22] and balance yin and yang. These herbs' comprehensive treatment of menopausal depression is by tonifying the kidney, decreasing mental anxiety, harmonizing qi and blood, and so on. The antidepressant effects of DSHH on stress after ovariectomization may also be related to changes in sex hormone levels. The multitarget effects of traditional Chinese medicine therapy have a great advantage in the treatment of depression.

## Figures and Tables

**Table 1:** Effect of DSHH on body weight of rats with menopausal depression $\left(\bar{x}\pm s,g\right)$.

Group	*N*	Before castration	After castration	After medication
Sham surgery	8	470.53 ± 5.49	474.32 ± 6.34	484.53 ± 5.63
Surgery	7	475.29 ± 6.15	466.31 ± 5.43	488.54 ± 6.84
Surgery/stress	8	473.49 ± 4.75	457.73 ± 4.15^∗^	474.32 ± 6.22^$^
Fluoxetine	7	479.15 ± 4.94	462.69 ± 5.28^∗^	478.33 ± 6.39^$^
DSHH	8	469.64 ± 5.23	452.74 ± 6.36^∗^	488.16 ± 5.54^$^

Compared with the sham surgery group, ^∗^*P* < 0.01, one way ANNOVA; ^$^*P* < 0.01, *t*-test, compared with after castration.

**Table 2:** Open-box experiment of menopausal depression rat $\left(\bar{x}\pm s\right)$.

Group	*N*	Before castration		After surgery/stress		After medication	
		Horizontal movement	Vertical movement	Horizontal movement	Vertical movement	Horizontal movement	Vertical movement
Sham surgery	8	54.3 ± 4.5	11.1 ± 2.5	45.2 ± 5.5^∗∗^	10.4 ± 3.7^∗∗^	48.4 ± 4.9^∗∗^	10.8 ± 2.4^∗∗^
Surgery	7	55.7 ± 5.3	10.9 ± 2.2	35.1 ± 4.8	8.2 ± 2.5	38.7 ± 3.8^∗^	8.4 ± 1.9^∗^
Surgery/stress	8	59.2 ± 6.2	11.8 ± 1.7	28.6 ± 5.3	7.5 ± 3.2	21.8 ± 3.9	7.4 ± 2.9
Fluoxetine	7	57.3 ± 8.6	9.8 ± 3.6	27.3 ± 5.2^∗^	5.1 ± 1.5^∗^	48.3 ± 2.5^∗∗^	9.7 ± 2.3^∗∗^
DSHH	8	52.4 ± 1.9	10.5 ± 1.9	21.3 ± 2.1^∗^	6.8 ± 2.9^∗^	50.2 ± 4.2^∗∗^	9.7 ± 2.9^∗∗^

Compared with the surgery/stress group, ^∗^*P* < 0.01 and ^∗∗^*P* < 0.001.

**Table 3:** Sucrose intake in rats with menopausal depression $\left(\bar{x}\pm s,g\right)$.

Group	*N*	Before castration	After castration/stress	After medication
Sham surgery	8	23.32 ± 3.53	21.84 ± 2.74^∗^	22.15 ± 2.26^∗∗^
Surgery	7	25.27 ± 3.37	16.44 ± 2.06^∗^	15.66 ± 2.83^∗∗^
Surgery/stress	8	22.54 ± 2.43	6.34 ± 1.65	7.14 ± 1.69
Fluoxetine	7	24.84 ± 2.68	8.85 ± 2.43	18.98 ± 2.68^∗∗^
DSHH	8	22.32 ± 3.05	7.45 ± 2.87	19.58 ± 2.22^∗∗^

Compared with the surgery/stress group, ^∗^*P* < 0.01 and ^∗∗^*P* < 0.001.

**Table 4:** Comparison of monoamine and cortisone levels in CSF $\left(\bar{x}\pm s\right)$.

Group	*N*	NE (pg/mL)	DA (pg/mL)	5-HT (pg/mL)	Cortisone (ng/mL)
Sham surgery	8	1.98 ± 1.23	4.98 ± 1.45^∗^	6.05 ± 1.13^∗^	0.36 ± 0.26^∗^
Surgery	7	1.88 ± 1.34	5.12 ± 1.78	5.98 ± 1.32	0.73 ± 0.53
Surgery/stress	8	1.33 ± 0.56	1.86 ± 1.48	3.56 ± 0.44	0.89 ± 0.35
Fluoxetine	7	1.98 ± 0.57^∗^	4.15 ± 1.88^∗^	4.47 ± 1.35	0.53 ± 0.26^∗^
DSHH	8	1.75 ± 0.76^∗^	4.26 ± 1.25^∗^	5.08 ± 1.36^∗^	0.45 ± 0.22^∗∗^

Compared with the surgery/stress group, ^∗^*P* < 0.05 and ^∗∗^*P* < 0.01.

**Table 5:** Comparisons of serum sex hormone levels in rats after manipulation $\left(\bar{x}\pm s\right)$.

Group	*N*	E2 (pg/mL)	FSH (mIU/mL)	LH (mIU/mL)
Sham surgery	8	5.39 ± 1.88^∗∗^	12.34 ± 1.46^∗∗^	17.54 ± 2.55^∗∗^
Surgery	7	1.87 ± 1.02	15.65 ± 1.44^∗∗^	24.98 ± 2.67
Surgery/stress	8	1.34 ± 1.25	23.57 ± 1.56	28.43 ± 4.35
Fluoxetine	7	2.45 ± 1.36^∗^	15.32 ± 1.22^∗∗^	18.33 ± 2.54^∗∗^
DSHH	8	2.76 ± 1.29^∗^	16.77 ± 1.46^∗∗^	17.95 ± 1.53^∗∗^

Compared with the surgery/stress group, ^∗^*P* < 0.01 and ^∗∗^*P* < 0.001.
